# Lignin-Based Spherical Structures and Their Use for Improvement of Cilazapril Stability in Solid State

**DOI:** 10.3390/molecules25143150

**Published:** 2020-07-09

**Authors:** Małgorzata Stanisz, Łukasz Klapiszewski, Dariusz T. Mlynarczyk, Beata J. Stanisz, Teofil Jesionowski

**Affiliations:** 1 Institute of Chemical Technology and Engineering, Faculty of Chemical Technology, Poznan University of Technology, Berdychowo 4, PL-60965 Poznan, Poland; malgorzata.m.stanisz@doctorate.put.poznan.pl (M.S.); lukasz.klapiszewski@put.poznan.pl (Ł.K.); 2 Chair and Department of Chemical Technology of Drugs, Poznan University of Medical Sciences, Grunwaldzka 6, PL-60780 Poznan, Poland; mlynarczykd@ump.edu.pl; 3 Chair and Department of Pharmaceutical Chemistry, Poznan University of Medical Sciences, Grunwaldzka 6, PL-60780 Poznan, Poland; bstanisz@ump.edu.pl

**Keywords:** biopolymers, spherical particles, surfactants, cilazapril, drug stability

## Abstract

Biopolymer-based spherical particles exhibit unique properties including narrow sizes and many functional groups on their surfaces. Therefore, they show great potential for application in many scientific and industrial processes. The main aim of this study was to prepare lignin-based spherical particles with the use of a cationic surfactant, hexadecyl(trimethyl)ammonium bromide (CTAB). In the first step, different preparation procedures were tested with varying parameters, including biopolymer and surfactant ratios, lignin filtration, and experimental time. The morphological and dispersion characteristics of the materials were determined to select the best samples with the most promising properties, which could then be tested for their acute toxicity. It was observed that almost all materials were characterized by spherical shapes in micro- and nanosizes. The sample with the best physicochemical properties was used for further analysis and then tested for medical applications: the improvement of the stability of a drug molecule, cilazapril (CIL). The formulated material (CIL@LC-2a 1:1 wt./wt.) exhibited outstanding properties and significantly improved the stability of cilazapril as tested in conditions of increased temperature and humidity. Lignin spherical particles may be employed as a promising material for shielding other active compounds from decomposition.

## 1. Introduction

Lignin is a naturally occurring polymer which can be found in plants [1]. It has a heterogeneous amphiphilic structure containing benzene rings as well as reactive aliphatic and phenolic hydroxyl and phenolic functional groups, and is characterized as a biocompatible, biodegradable, and renewable material [1,2,3,4]. To date, this biopolymer has been widely used as a heating source to obtain energy, mostly for cellulose and hemicellulose-based industries, in which it is a waste product [5]. The presence of reactive functional groups enables the chemical modification of its structure and therefore extension of its scope of application to high-value materials such as abrasive tools [6,7], adsorbents [8,9], binders, dispersants, biofuels, and biochemicals [10,11]. Recently, it has been shown that lignin can be considered as an efficient material for the production of micro- and nanospheres [12,13,14]. Lignin-based spherical structures exhibit low toxicity, favorable parameters of controlled release, biocompatibility and biodegradability [15,16,17]. They are especially used as absorbents of ultraviolet radiation, harmful ions and other pollutants, biocatalysts and drug carriers [18,19,20,21,22]. Moreover, the biopolymer-based spherical structures can enhance antibacterial and antioxidant properties, and are also tested for applications in the preparation of coatings, tissue engineering, nanoscience, and nanotechnology [16,23,24,25]. The preparation of biopolymer-based structures is mostly simple and inexpensive, and can be performed on an industrial scale. During the synthesis, the size and morphology of the particles can be controlled [16].

Lignin-based spherical structures can be prepared with the use of a soft-templating method. In this procedure, emulsion droplets, surfactant micelles or gas bubbles can be employed [26]. One of the frequently used substances is the cationic surfactant hexadecyl(trimethyl)ammonium bromide (CTAB). CTAB has already been applied as a soft template for the preparation of silica [27,28,29] and alumina [30], and there are also indications that it could be used for the synthesis of biopolymer-based spherical particles [29,31,32]. Most commonly, lignosulfonates have been employed as substances in the self-assembly process. Tang et al. prepared colloidal spheres with different CTAB and sodium lignosulfonate (LS) ratios. They reported that the water content is very important for the assembly of spherical structures, and that the morphology and size of the resulting particles depend on the amount of the solvent added to the system [33]. Peng et al. obtained sodium lignosulfonate and CTAB spheres in which the drug avermectin was loaded. The prepared formulations exhibited controlled release profiles, and their physical and chemical stability was good at low and high temperatures [34]. There exist other publications regarding lignosulfonate combined with CTAB particles loaded with avermectin [35]. Moreover, Li et al. reported on drug-loaded lignin-based particles which exhibited anti-photolysis properties and excellent sustained release [36]. 

The stability of drugs is a very important factor in disease treatment. Sometimes it needs to be improved for better substance activity [37]. This is particularly true in the case of drugs such as cilazapril (CIL), which is a prodrug that belongs to the class of angiotensin enzyme inhibitors and is widely prescribed for hypertension and heart failure. It has been previously shown that the stability of cilazapril decreases especially in open containers during exposure to air humidity and high temperatures [38]. The degradation of cilazapril results in a decrease in its bioavailability, as it is transformed into cilazaprilat, a product of an ester hydrolysis process [38]. It is also worth noting that certain commonly used excipients, such as lactose and talc, negatively affect the stability of cilazapril. It has been reported that polyvinylpyrrolidone (PVP) stabilizes cilazapril and may potentially be used for the preparation of dosage forms [39]. However, it is important to develop other simple methods for improving cilazapril stability.

In the present study, lignin-based spherical particles were fabricated with the use of different synthesis methods. The ratios of precursors, process time and filtration of lignin were studied. Microstructural and dispersion analysis were performed to select the best system prepared with the use of the biopolymer. Lignin-based spherical particles were applied as a stabilizer for the cilazapril prodrug, which, to the best of our knowledge, has not been done previously. The obtained structures were ground mechanically with the active substance to prepare a homogeneous system. Moreover, extended physicochemical and microstructural analysis as well as stability tests were performed to confirm the effectiveness of the proposed procedure. 

## 2. Results and Discussion

### 2.1. Characteristics of Lignin-CTAB (LC) Spherical Particles

#### 2.1.1. Morphological and Dispersion Properties 

The aim of this study was to prepare lignin-based spherical structures with the use of a cationic surfactant, hexadecyl(trimethyl)ammonium bromide. The best structure was further applied for improvement of the stability of the active compound cilazapril. It was therefore important to compare all of the materials prepared with the use of three different methods, and then select the best one. In the first method, kraft lignin was dispersed in ethyl alcohol and then mixed with an ethanolic surfactant solution for 2 h (process 2a) or 4 h (process 4). In the second approach (process 2b), the lignin dispersion, after being stirred in ethanol, was filtered before the CTAB solution was added. The process then continued as in process 2a. In each process, the following ratios of lignin to CTAB were tested: 1:1 wt./wt., 2:1 wt./wt., and 4:1 wt./wt.

**Figure 1:**
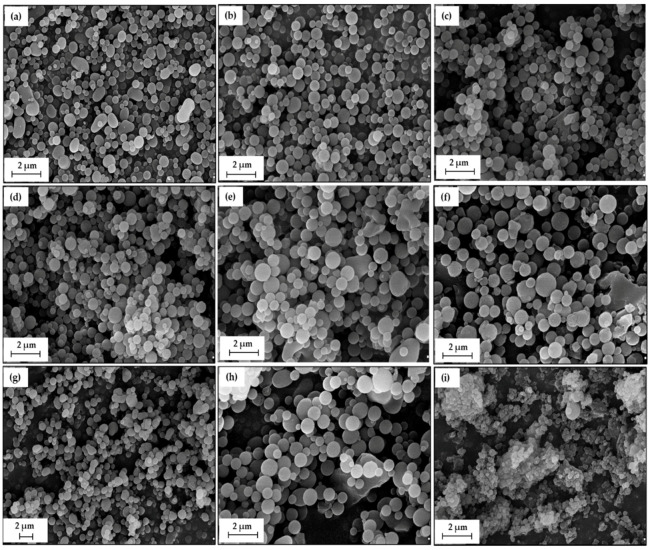
SEM images of LC-2a (1:1 wt./wt.) (**a**), LC-2a (2:1 wt./wt.) (**b**), LC-2a (4:1 wt./wt.) (**c**), LC-2b (1:1 wt./wt.) (**d**), LC-2b (2:1 wt./wt.) (**e**), LC-2b (4:1 wt./wt.) (**f**), LC-4 (1:1 wt./wt.) (**g**), LC-4 (2:1 wt./wt.) (**h**) and LC-4 (4:1 wt./wt.) (**i**).

Morphological and microstructural properties of the obtained materials are very significant for further experiments and applications. As shown in Figure 1, SEM images of all products were prepared. Moreover, their size distributions by volume, maximum volume contributions, and polydispersity indices are given in Table 1.

Lignin-based spherical particles can be prepared with the use of all of the presented methods. All of the samples were characterized by spherical or ellipsoidal shape (see Figure 1a). According to the particle size analysis (see Table 1) almost all of the particles were in the range of sizes smaller than 1 µm, with the exception of LC-2b (4:1 wt./wt.), which appeared in two particle size ranges (106–1106 nm and 4145–6439 nm), with maximum volume contributions of 9.2% and 12.4% coming from diameters 295 and 5560 nm respectively. On the other hand, the smallest particles (220–531 nm) were recorded for LC-2a (2:1 wt./wt.), as may also be observed in the corresponding SEM image (Figure 1b). The maximum volume contribution of 27.1% comes from particles of 342 nm in diameter. In structures prepared with the use of process 4, particles of 396 nm give the maximum volume contribution, equal to 27.8%, 24.5%, and 20.8% for LC-4 (1:1 wt./wt.), LC-4 (2:1 wt./wt.), and LC-4 (4:1 wt./wt.) respectively. All products exhibit relatively low polydispersity indices (PdI < 0.35), which also confirms the correctness and effectiveness of the described experiments. The particle size distributions according to volume obtained for all samples are presented in the Supplementary Figure S1. Apart from sample LC-2b (4:1 wt./wt.), all of the analyzed materials were characterized by one relatively narrow band covering the particle diameters.

Products prepared with the use of the biopolymer and surfactant in the ratio 1:1 were the most homogeneous, and no residue of the precursor material was observed. Some irregular-shaped particles can be observed for the obtained materials, especially at precursor ratios of 2:1 wt./wt. and 4:1 wt./wt. (Figure 1e,f), as well as in the product obtained after longer process duration (Figure 1h,i). It appears that the technique of addition of lignin and longer mixing of precursors influence the binding process of the biopolymer and surfactant. It was also noted that longer mixing and lower addition of surfactant lead to an increased ability to form aggregates and agglomerates (see Figure 1i).

#### 2.1.2. Acute Toxicity Assessment 

Following an assessment of the morphological and dispersion characteristics of the obtained materials, acute toxicity evaluation using the Microtox*^®^* test was performed for the most promising materials. LC-2a (1:1 wt./wt.), LC-2b (2:1 wt./wt.), LC-4 (1:1 wt./wt.) and LC-4 (2:1 wt./wt.) were selected for further examination, as they exhibited at least one of the following properties: the smallest particle sizes, the lowest PdI, or the lowest tendency to form aggregates and agglomerates.

The Microtox*^®^* test is based on the measurement of *Aliivibrio fischeri* bioluminescence changes, which correspond to cell viability [40]. The acute toxicity screening evaluation of the material is useful for the assessment of chemical compounds and materials [40,41,42]. The bacterial cultures were exposed to suspensions of the materials for 15 min, with measurements of bioluminescence made before exposure and after 5 and 15 min of exposure. Inhibition of metabolism by 20% or lower is regarded as non-toxic [40].

As can be seen in Table 2, the samples LC-2a (1:1 wt./wt.) and LC-4 (1:1 wt./wt.) did not exhibit a toxic effect after 5 min of the experiment. The other two samples, LC-2b (2:1 wt./wt.) and LC-4 (2:1 wt./wt.), decreased *A. fischeri* cell viability more significantly, especially LC-2b (1:1 wt./wt.), which inhibited the metabolism of bacteria the most out of all four samples (33%). This result might be associated with the different method of preparation of lignin solution compared with other samples and with different bonding of kraft lignin and CTAB. Moreover, after an additional 10 min, it was observed that only LC-2a (1:1 wt./wt.) did not exhibit acute toxicity. Based on these results, lignin-based spherical structures with a 1:1 ratio of biopolymer and surfactant were found to exhibit the best properties and were selected for further examination and combination with the active compound cilazapril.

### 2.2. Characterization of CIL@LC-2a (1:1 wt./wt.) Material

#### 2.2.1. Morphological and Dispersion Properties

**Figure 2:**
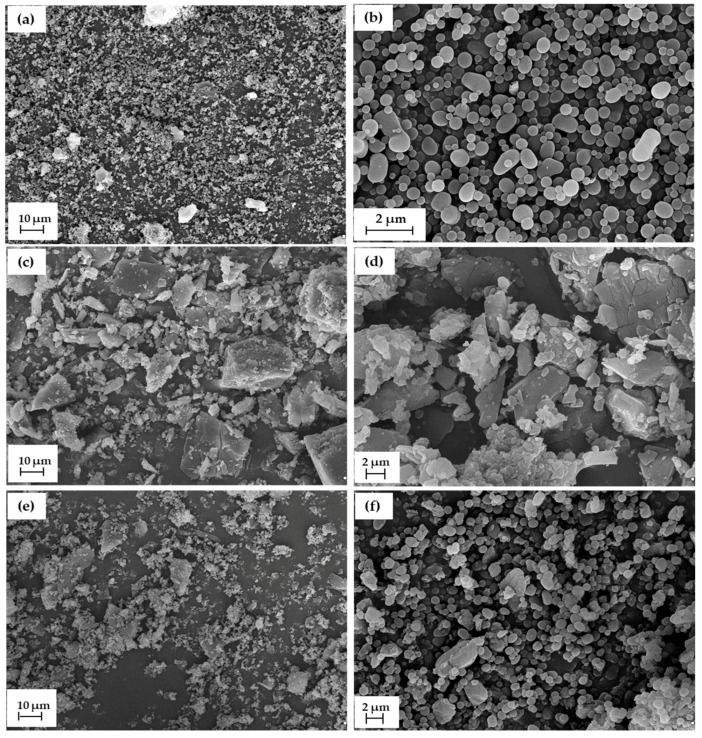
SEM images of LC-2a (1:1 wt./wt.) (**a**,**b**), CIL (**c**,**d**) and CIL@LC-2a (1:1 wt./wt.) (**e**,**f**); shown in two magnifications.

Characterization of the dispersion and morphology of lignin-based spherical particles, cilazapril and CIL@LC-2a (1:1 wt./wt.) was performed with the use of scanning electron microscope images (see Figure 2), as well as particle size distributions by volume, maximum volume contributions, and polydispersity indices (see Table 3).

The SEM images and dispersion properties of LC-2a (1:1 wt./wt.) have already been given in the previous section, but for clearer characterization of the final products they are also included in Figure 2. LC-2a (1:1 wt./wt.) particles exhibited high homogeneity with spherical and partially ellipsoidal structures. Their sizes ranged from 295 to 825 nm, with a low polydispersity index (0.186) and a maximum volume contribution of 28.3% from particles of 531 nm diameter. On the other hand, cilazapril particles exhibited irregular shape and small size (38–68 nm), although there were also larger particles, up to 615 nm, with maximum volume contributions of 12.1% and 20.4% for structures of 51 nm and 164 nm respectively. It should be noted that the material CIL@LC-2a (1:1 wt./wt.) included both particle types, i.e., larger drug particles and smaller biopolymer-based spheres, after mechanical grinding, which confirms the correctness of the procedure used. As expected, the sample showed a tendency to form larger agglomerates. Therefore, the particle sizes ranged from 164 to 3091 nm, with a maximum volume contribution of 12.2% for materials of sizes 1718 nm and 1990 nm. Moreover, the polydispersity index was much lower for CIL@LC-2a (1:1 wt./wt.) (0.363) than for pure cilazapril (0.821). It is also noted that the shape of lignin-based spheres remained intact. The smaller biopolymeric particles were visible on larger drug structures. The presence of larger particles of cilazapril leads to a less homogeneous structure of the final material. The particle size distributions by volume are also presented in the Supplementary Figure S1.

#### 2.2.2. Fourier Transform Infrared Spectroscopy (FTIR)

**Figure 3:**
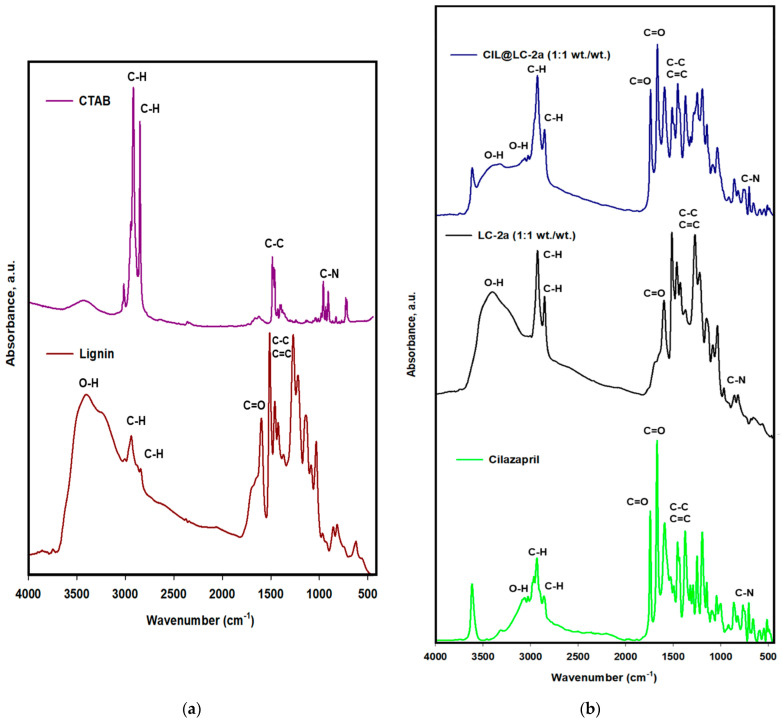
Fourier transform infrared spectra of lignin and CTAB (**a**), cilazapril, LC-2a (1:1 wt./wt.) and CIL@LC-2a (1:1 wt./wt.) (**b**).

FTIR spectra of kraft lignin, CTAB, pure cilazapril, lignin-based spherical particles and their combination with the active compound were analyzed to assess the correctness and effectiveness of the synthesis. All FTIR spectra are presented in Figure 3, and the wavenumbers of characteristic bands for all samples are indicated.

Kraft lignin (L), one of the organic precursors used for preparation of the spherical particles, consists of three basic phenylpropanoid units, which possess a variety of functional groups. This biopolymer is characterized by the presence of stretching vibrations of O–H bonds with a maximum at wavenumber 3400 cm^−1^ and stretching vibrations of C–H bonds at the wavenumbers 2950 cm^−1^ and 2800 cm^−1^. Moreover, characteristic bonds for kraft lignin also include C=O (1600 cm^−1^), C–C and C=C (1500–1350 cm^−1^) which are associated with aromatic rings present in the biopolymeric structure. In addition, there are C–O(H) and C–O(Ar) bands at 1250–1150 cm^−1^, as well as C–O–C observed with a maximum at wavenumber 1000 cm^−1^. The obtained absorption maxima are consistent with the spectra for lignin obtained in previous studies [9,43,44,45,46].

As a soft template, hexadecyl(trimethyl)ammonium bromide (CTAB) was used. CTAB is characterized by the presence of stretching vibrations of C–H bonds with maximum adsorption recorded at wavenumbers of 2900 cm^−1^ and 2850 cm^−1^ and stretching vibrations of C–C bonds with a maximum at 1500 cm^−1^. Moreover, very significant stretching vibrations of C–N bonds were observed with a maximum at wavenumber 3050 cm^−1^ and at wavenumbers of 900–750 cm^−1^ [47,48,49].

Cilazapril, an active compound, is also characterized by many signals, as shown in Figure 3. The band at 3300 cm^−1^ corresponds to N–H stretching vibrations. In turn, a broad band associated with O–H bands was shifted to smaller wavenumbers, appearing at frequencies between 3100 and 3000 cm^−1^. Stretching vibrations of C–H bonds were observed with maxima at wavenumbers of 2950 cm^−1^ and 2900 cm^−1^. Bands with high intensity associated with stretching vibrations from carbonyl functional groups were recorded at 1750 cm^−1^ and 1650 cm^−1^. There are also bands at wavenumbers 1600–1450 cm^−1^ corresponding to C–C and C=C bonds, at frequencies in the range 1250–1200 cm^−1^ associated with C–O(H) and C–O(Ar) bonds, and at 1200 cm^−1^ corresponding to C–O–C. There is also an important signal in the wavenumber range 1100–950 cm^−1^, which confirms the presence of C–N bonds [38,39]. Additionally, with a maximum at 3600 cm^−1^, there is a band which may be associated with chained H_2_O molecules without hydrogen bonds in the crystal lattice of cilazapril structural O–H groups. 

FTIR spectra of the synthesized products were also obtained. The spectrum of LC-2a (1:1 wt./wt.) corresponds in most cases with the spectrum of pristine kraft lignin. There are only a few changes which confirm the correctness of the described process. The C–H bands with maxima at wavenumbers 2950 cm^−1^ and 2800 cm^−1^ increased in intensity. This may be due to the influence of CTAB on the final product. Moreover, there is also a band with a maximum within the range 950–900 cm^−1^, which appeared in the final structure after addition of the surfactant.

The FTIR spectrum of the material containing cilazapril mixed with LC-2a (1:1 wt./wt.) confirmed the effectiveness of the mechanical grinding process. This spectrum includes all of the bands corresponding to LC-2a (1:1 wt./wt.), but most importantly those corresponding to pristine cilazapril. There is also a band with a maximum at 3600 cm^−1^, which might be associated with water attached to the drug. In comparison with LC-2a (1:1 wt./wt.), the final sample produces broad bands associated with O–H bonds, which appear at 3400 cm^−1^ and in the range 3100–3000 cm^−1^. Moreover, there are bands with maxima at wavenumbers 1750 cm^-1^ and 1650 cm^-1^, which are associated with carbonyl functional groups. All other bands are at similar frequencies, as in the case of cilazapril and LC-2a (1:1 wt./wt.). 

#### 2.2.3. Zeta Potential

**Figure 4:**
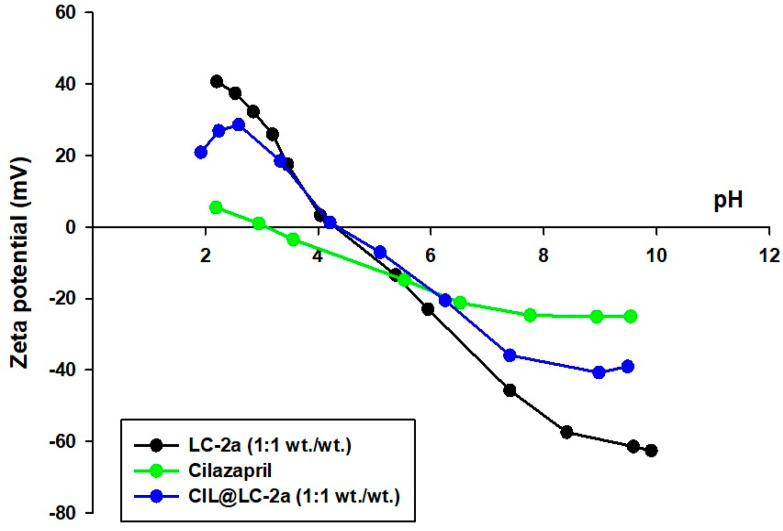
Zeta potential vs. pH for LC-2a (1:1 wt./wt.), cilazapril and CIL@LC-2a (1:1 wt./wt.).

The electrokinetic curves for LC-2a (1:1 wt./wt.), cilazapril and CIL@LC-2a (1:1 wt./wt.), which show the influence of pH on the electrokinetic stability of the products, are presented in Figure 4. The measurements were carried out at pH ranging from 2 to 10. Samples can be classified as electrokinetically stable if their zeta potential exceeds 30 mV or is below −30 mV. During this analysis, the surface charge of the obtained systems can be evaluated.

During the formation of lignin-based spherical particles, anionic biopolymer groups were electrostatically attracted to cationic groups from CTAB [33,36]. 

Based on the cilazapril electrokinetic curve obtained in this study, it was estimated that the active compound does not exhibit electrokinetic stability in the entire pH range. The lowest value (–25 mV) for the sample was recorded at pH = 8. The pH_IEP_ value for cilazapril was 3.1. 

On the other hand, LC-2a (1:1 wt./wt.) exhibited high stability at pH values below 2.5 and above 7. It attained its isoelectric point at pH = 4.0. At IEP the surface negative charges of lignin were neutralized by CTAB, and the hydrophilicity of the compound is the lowest and the hydrophobicity the highest [33]. The IEP for the sample CIL@LC-2a (1:1 wt./wt.) also occurs at the same pH value as for the lignin spherical particles LC-2a (1:1 wt./wt.); compared to CIL the values were shifted from 3.1 to 4.0. The shift of the IEP towards higher pH in the case of CIL@LC-2a (1:1 wt./wt.) may be caused by the ionization effect of CTAB. It has been previously shown that lignin has no isoelectric point in the analyzed pH range, which may be related to the presence of hydrophilic groups such as phenolic hydroxyl and carboxylate groups [50]. The IEP of CTAB has been calculated at pH = 9.8 (data not presented in Figure 4). CTAB shifted the electrokinetic values of lignin in acidic conditions. It may be observed that combination with LC-2a (1:1 wt./wt.) improved the electrokinetic stability of cilazapril, and the material was electrokinetically stable at pH above 7. Moreover, the zeta potential of all samples changes from positive to negative with an increase in pH. 

#### 2.2.4. Thermogravimetric Analysis

**Figure 5:**
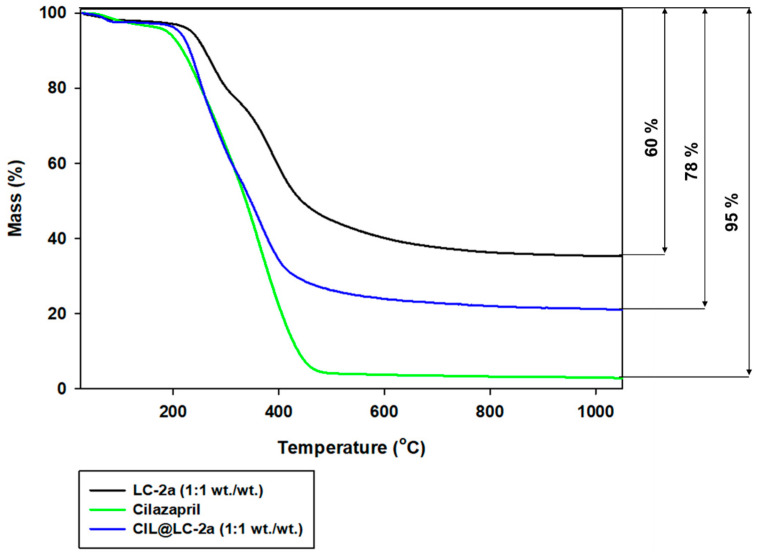
TGA curves of LC-2a (1:1 wt./wt.), cilazapril and CIL@LC-2a (1:1 wt./wt.).

Thermal stability is an important factor for determination of the influence of lignin-based spherical structures on the active compound cilazapril. To assess this parameter, thermogravimetric analysis was performed for cilazapril, LC-2a (1:1 wt./wt.) and their combination. The results are presented in Figure 5.

Cilazapril exhibited very limited thermal stability, and lost almost 95% of its initial mass above 400 °C. The first stage of mass loss (at temperatures up to 200 °C) is associated with desorption of physically bound water. At higher temperatures, cilazapril decomposes rapidly [38,39]. This results from the presence of the ester functional group, which is very reactive and sensitive to high temperatures as well as hydrolysis. Water bound in the crystal lattice of CIL with increased temperature accelerated the hydrolysis of this compound. 

LC-2a product (1:1 wt./wt.) also showed limited thermal stability, but it was more stable at higher temperatures than cilazapril. It lost up to 60% of its initial mass above 600 °C. There are three characteristic stages, which are associated with mass loss of lignin-based compounds at increased temperatures. First, as in the case of cilazapril, the loss of physically bound water occurs (up to 200 °C). Next, lignin undergoes partial depolymerization and disruption of aliphatic bonds (in the range 220–600 °C). Finally, the breakdown of the aromatic parts of lignin and its fragmentation are visible at temperatures above 600 °C. The structure of lignin is very complex, and the degradation products depend strongly on the type of biopolymer, the bonds linking the monomers, and the moisture content. These data correlate well with those reported in previous studies [51,52].

As can be observed in the thermogravimetric curve of CIL@LC-2a (1:1 wt./wt.), the addition of the lignin-based spherical structures LC-2a (1:1 wt./wt.) to CIL improves the thermal stability of the final product. The total mass loss for the obtained material was equal to 78% in the analyzed temperature range.

### 2.3. Stability Assessment of CIL@LC-2a (1:1 wt./wt.) Material

#### 2.3.1. Selection and Validation of the Analytical Method

Validation of the reversed phase high-performance liquid chromatography (RP-HPLC) method was performed for qualitative and quantitative determination and to provide a rapid assessment procedure for degradation of CIL@LC-2a (1:1 wt./wt.) samples. The presented method was previously applied for the determination of cilazapril in pure [38] and other ACE-Is [53,54,55], which are structurally related. 

The following validation parameters were determined in this study: selectivity, sensitivity (limit of detection–LOD and limit of quantification–LOQ), precision and linearity.

**Figure 6:**
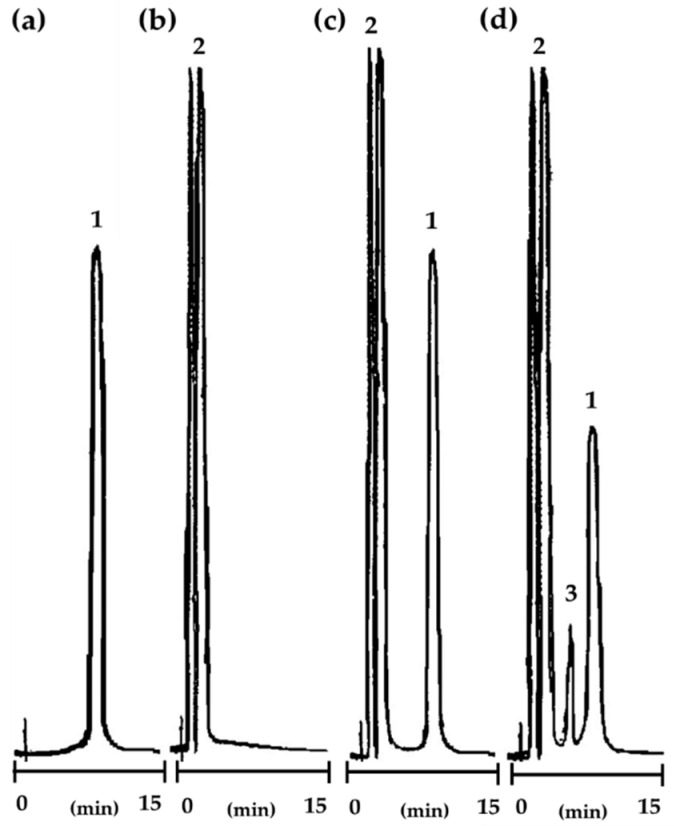
RP-HPLC chromatograms for: cilazapril in pure (**a**), LC-2a (1:1 wt./wt.) (**b**), CIL@LC-2a (1:1 wt./wt.) before degradation (**c**) and CIL@LC-2a (1:1 wt./wt.) after degradation (**d**). Retention times: cilazapril (1): t_R_ ~ 8 min; LC-2a (1:1 wt./wt.) (2): t_R_ ~ 2–4 min and degradation product of cilazapril (3): t_R_ ~6 min.

The validation report is presented in Figure 6, and also in Supplementary Table S1. Chromatograms are given for cilazapril in pure, LC-2a (1:1 wt./wt.), and CIL@LC-2a (1:1 wt./wt.).

In the analysis of the chromatograms, well-separated and sharply developed peaks were observed. In addition, reasonable retention times of selected peaks indicated the good selectivity of the method. Signals of LC-2a (1:1 wt./wt.) were obtained in a time range of 2–4 min, and in the case of cilazapril in pure signals were obtained at 8 min. The differences in retention times of cilazapril and lignin-based spherical particles indicate no interference between the biopolymer substance, the active compound, and its degradation product. The correlation coefficient of the calibration curve was high, at r = 0.999, and indicated that the method was linear in the analyzed concentration range from 0.04 mg/mL to 0.4 mg/mL. Peak 3 in Figure 6d can be associated with cilazaprilat, the degradation product of cilazapril. It is formed during the de-esterification reactions and has a negative impact on pharmaceutical availability. The degradation of the active compound usually occurs during the hydrolysis of carbonyl functional groups [38].

The precision of the proposed method was acceptable for further kinetic analysis. It was observed that, for the mean recovery of CIL@LC-2a (1:1 wt./wt.), the intra-day precision and inter-day precision were 100.18 ± 0.49% and 100.20 ± 0.63% respectively. Moreover, the sensitivity was also satisfactory, with values of 0.023 mg/mL for LOD and 0.070 mg/mL for LOQ.

#### 2.3.2. Stability Studies of Cilazapril in Pure and CIL@LC Formulation

Thermodynamic and kinetic parameters of cilazapril degradation from cilazapril in pure and CIL@LC-2a (1:1 wt./wt.) are given in (Supplementary Table S2). After exposure of the sample to increased temperature ranges at elevated relative humidity (RH), at different sampling times, the remaining amount of drug was used for calculation of the kinetic order of degradation of CIL@LC-2a (1:1 wt./wt.). It was also noted that the reaction was characterized by first-order kinetics, and only one degradation product was observed. The calculation of degradation rate constants was performed with the use of the formula presented below (Equation (1)):

(1) $$\ln c_{t}=\ln c_{0}-\mathrm{kt}$$

where $c_{t}\left(\%\right)$ is the concentration of cilazapril in the sample at time t (h), $c_{0}$ is the initial concentration of cilazapril at time t_0_, and k (1/s) is the reaction rate constant.

**Figure 7:**
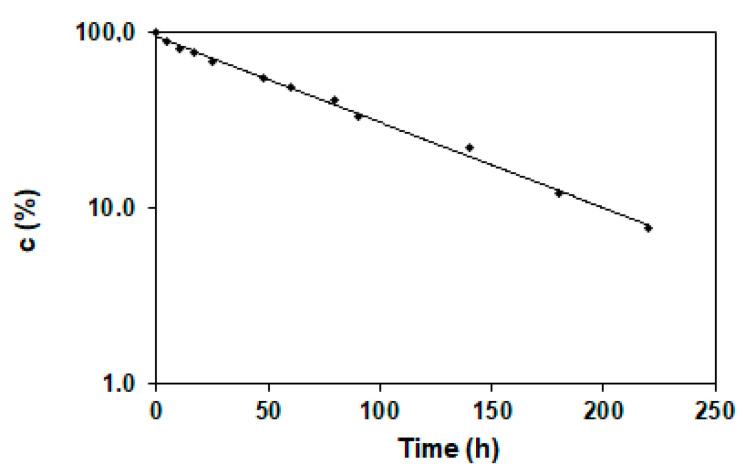
Semilogarithmic plot of the degradation of CIL@LC-2a (1:1 wt./wt.) product at 90 °C and humidity 76.4%.

An example semilogarithmic plot c = f(t) is shown in Figure 7. As predicted, the plot was linear and the slope corresponded to the magnitude of the degradation rate, which is characteristic for first-order kinetics.

**Figure 8:**
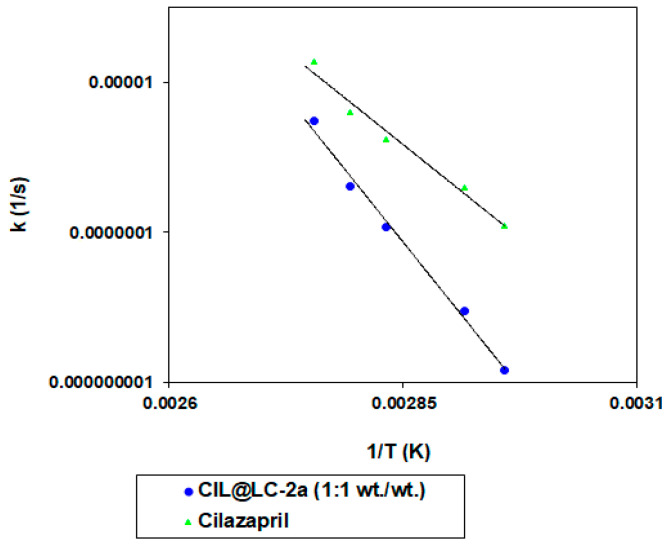
Semilogarithmic linear dependence of the reaction rate constant and the inverse of temperature for stability evaluation of cilazapril in pure and CIL@LC-2a (1:1 wt./wt.).

The activation energy (E_a_), which was calculated for cilazapril in pure and CIL@LC-2a (1:1 wt./wt.), describes the strength of cleaved bonds in selected molecules during degradation. As can be seen in Figure 8, the formulation of cilazapril with lignin-based spherical particles had a higher activation energy (303.79 ± 62.51 kJ/mol) than its pure form (166.49 ± 20.83 kJ/mol). More detailed data can be found in Supplementary Table S2. It can be concluded that, with an increased E_a_ value and lower k value, the described product is more stable, and is therefore safer for use after a prolonged period of time. It is also indicated that the addition of lignin-based spherical particles improved the stability of cilazapril relative to its pure form.

The effect of humidity on the stability of CIL@LC-2a (1:1 wt./wt.) was determined at 90 °C, with relative humidity in the range 25.0–76.4%. The results are given in Table 4.

It was observed that the cilazapril formulation with lignin-based spherical particles has a lower slope (0.0304 ± 0.0068) than the pure active substance (0.0361 ± 0.0062). This indicates that the studied substance exhibits lower sensitivity to humidity. The ester functional group present in the CIL structure is very reactive in humid reaction conditions. It can be concluded that lignin-based spherical particles have a protective effect on cilazapril molecules.

## 3. Materials and Methods 

### 3.1. Materials

Kraft lignin (Sigma-Aldrich, Steinheim, Germany), hexadecyl(trimethyl)ammonium bromide (Sigma-Aldrich, Steinheim, Germany) and ethyl alcohol were used to prepare lignin-based spherical structures. The particles were obtained with the use of three different lignin-to-CTAB ratios: (i) 1 part by weight of kraft lignin per 1 part by weight of surfactant (1:1), (ii) 2 parts by weight of kraft lignin per 1 part by weight of surfactant (2:1), and (iii) 4 parts by weight of kraft lignin per 1 part by weight of surfactant (4:1). 

Cilazapril monohydrate (Biofarm; serial number 1621816, Poznan, Poland) was used to prepare the final products. Methanol and acetonitrile (Merck, Darmstadt, Germany) were of HPLC grade. Monobasic potassium phosphate, sodium chloride, sodium nitrate, sodium bromide, potassium iodide, sodium iodide, and orthophosphoric acid (Merck, Darmstadt, Germany) were also used. All reagents and solvents were used as supplied without further purification.

### 3.2. Preparation of Lignin-CTAB (LC) Spherical Particles

Biopolymer-based spherical particles were prepared with the use of a cationic surfactant as a soft template. Kraft lignin (2 g) was dispersed with high-speed stirring (800 rpm) in 50 mL of ethyl alcohol for 1 h. Meanwhile, CTAB (2 g, 1 g, or 0.5 g) was also dissolved in 50 mL of ethyl alcohol. The surfactant was then added to the lignin dispersion, and the solution was mixed for 2 h (process 2a) or 4 h (process 4). Then, the lignin-CTAB mixture was filtered and 500 mL of deionized water was added to the clear solution with the use of a peristaltic pump (10 mL/min). The obtained particles underwent vacuum filtration (MC membrane filter with 47 mm diameter and 0.22 µm pore size). 

**Figure 9:**
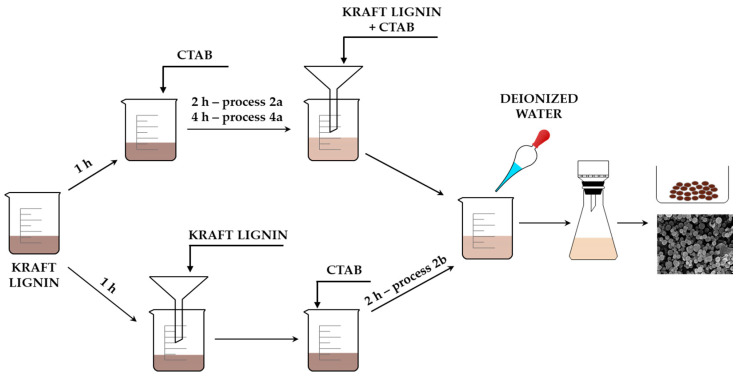
Schematic diagram of preparation of lignin-based spherical particles.

Lignin particles were also prepared with a modification of the procedure. The same amounts of precursors were used for this synthesis as for processes 2a and 4. The dispersion of kraft lignin in 50 mL of ethanol was first filtered, and ethanolic solution of CTAB was then added to the clear solution. It was mixed for 2 h (process 2b), and the same amount of deionized water as before was added to the mixture with the use of a peristaltic pump. The prepared material was filtered using a vacuum filtration system. The yield of particles was estimated at 90%. Methods of preparation are presented in Figure 9.

### 3.3. Preparation of CIL@LC Material 

Following detailed characterization of LC materials prepared using different process conditions, the material LC-2a (1:1 wt./wt.) was selected for further examination. LC particles were combined by means of a mechanical method which has been used in previous studies [51,52]. Appropriate amounts of LC (150 mg) and cilazapril monohydrate (150 mg) (1:1 wt./wt.) were ground together in a ball mill. Cilazapril is characterized by an ester functional group, which is very sensitive to water and may easily hydrolyze. During the formation of the lignin-based spherical particles, 500 mL of water is added to the system, which may be damaging for the active compound. Therefore, a mechanical method was used for combining the obtained particles with cilazapril. A homogeneous mixture was obtained after 30 min of constant mixing. 

### 3.4. Characterization of Biopolymer-Based Spherical Materials 

Physicochemical and morphological-dispersion analysis were performed for the obtained spherical structures, the active compound, and the final biopolymer-drug blend. 

The shape, size and surface morphology of particles were evaluated with the use of scanning electron microscopy (SEM). Images of the materials were obtained using an EVO40 microscope (Zeiss, Jena, Germany). For sample preparation, before testing, all materials were coated with Au for a time of 5 s using a Balzers PV205P coater (Oerlikon Balzers Coating SA, Brügg bei Biel, Switzerland). Additionally, particle size distributions and the polydispersity index were calculated using a Zetasizer Nano ZS instrument (Malvern Instruments Ltd., Malvern, UK), which operates based on the non-invasive backscattering (NIBS) technique, and is capable of measuring particle sizes in the range 0.6–6000 nm. Samples were dispersed in propan-2-ol with the use of ultrasonic bath Sonic-3 (Polsonic, Warsaw, Poland) for 5 min (Cp = 0.05%). Moreover, the polydispersity index (PdI) was calculated with the use of cumulant analysis (the fit of a polynomial to the log of the G1 correlation function), which may be presented as Equation (2) [56]:

(2) $$\ln\left[G1\right]=a+\mathrm{bt}+{\mathrm{ct}}^{2}+{\mathrm{dt}}^{3}+{\mathrm{et}}^{4}+\ldots$$

where b is the second order cumulant or the z-average diffusion coefficient, and c is the coefficient of the squared term, known as polydispersity when scaled as ${2c/b}^{2}\;$.

Acute toxicity assessment of selected samples was performed using a Microtox*^®^* acute toxicity test: the 81.9% screening test with the use of Microtox*^®^* M500 equipment with *Modern Water* MicrotoxOmni 4.2 software according to the manufacturer’s protocols (ModernWater plc) [40]. The decrease in *A. fischeri* cell viability was calculated according to the changes in the bioluminescence emitted by the bacteria as measured with Microtox*^®^* M500 with Modern Water MicrotoxOmni 4.2 software. The bacteria used in this test (*A. fischeri*) supplied by the producer are lyophilized, so that their cell walls are broken, which greatly increases their susceptibility to any toxins. In this way, it is possible to rapidly measure cell toxicity. However, the test samples are thermostated at 15 °C, at which temperature the bacteria are unable to survive for a longer period of time, as they are obtained from the skin of arctic squids. At 5 °C the bacteria survive 3 h. Attempting to adapt the procedure to lower the temperature of the test would require the validation of the method, as well as the development of additional equipment for the measurements. The Microtox test is a world standard in assessment of water toxicity, but it has also been found useful in initial assessment of nanoparticle and material toxicity [41,42].

Evaluation of the efficiency and correctness of the preparation of spherical particles and of the blend of lignin-based material with active compound, and determination of their functional groups, were carried out with the use of Fourier transform infrared spectroscopy (FTIR) in the wavenumber range 4000–450 cm^−1^. IR spectra were recorded using a Vertex 70 spectrometer (Bruker Optics GmbH, Ettlingen, Germany). All materials were prepared in the form of tablets, which consisted of 1 mg of analyzed material and approximately 250 mg of anhydrous KBr pressed in a steel ring at a pressure of 10 MPa.

The zeta potential of all materials was determined by the electrophoretic light scattering method with the use of a Zetasizer Nano ZS instrument, equipped with an autotitrator (Malvern Instruments Ltd., Malvern, UK). The samples were first dispersed in 0.001 M NaCl solution, and then the zeta potential was determined over a pH range of 2–10.

Thermal stability analysis of the obtained products was performed with the use of a Jupiter STA 449F3 analyzer (Netzsch GmbH, Selb, Germany) based on the thermogravimetric method (TGA), obtaining the dependence between the sample mass and temperature. All samples were weighed at approximately 10 mg, placed in an Al_2_O_3_ crucible, and heated in a nitrogen atmosphere at a rate of 10 °C/min from 30 to 1100 °C.

### 3.5. Selection of Analytical Method for Kinetic Studies of Cilazapril and CIL@LC Particles

Stability tests were evaluated with the use of the reversed phase high-performance liquid chromatography method, in which a high-performance liquid chromatograph (Shimadzu, Kyoto, Japan) was employed. The apparatus consisted of a Shimadzu LC-6A Liquid Chromatograph pump with a 7725 Rheodyne value injector (20 µL) and a Shimadzu SPD-6AV UV-VIS spectrophotometric detector set at 212 nm. The peak areas were integrated using the Shimadzu C-R6A chromatopac integrator. Columns (LiChroCART^®^ 250-4 mm HPLC-Cradridge, LiChrospher^®^ 100 RP-18 (5 µm), Merck, Darmstadt, Germany) operated at ambient temperature and were eluted at a flow rate of 1.5 mL/min. The mobile phase consisted of acetonitrile, methanol, and phosphate buffer (pH 2.0) (60:10:30, *v*/*v*/*v*).

First, the proposed method was validated. For this purpose, a stock solution was prepared on the day of the analysis by dissolving 20 mg of cilazapril in 50 mL of methanol, protected from light and stored at 2 °C before use. It was noted that, after 7 days of storage, it showed no evidence of decomposition. Next, the stock solution was diluted with methanol to prepare standard solutions ranging from 0.04 mg/mL to 0.4 mg/mL. Then, the calibration procedure for the HPLC analysis was performed. The calibration curve was determined by linear regression for cilazapril in pure and CIL@LC-2a (1:1 wt./wt.). Standard solutions of pure CIL and the obtained product were prepared at ten concentrations: 0.04, 0.08, 0.12, 0.16, 0.20, 0.24, 0.28, 0.32, 0.36, 0.40 mg/mL (C_i_). Each 25 µm of the standard solution was injected for RP-HPLC analysis, and the procedure was repeated three times under the conditions described above. The calibration curve was obtained by plotting the relative peak areas (P*_i_*_CIL_) versus the corresponding concentrations. The regression equation was computed with the use of the least squares method. 

Relative standard deviation (RSD) of replicate measurements was used for expression of the method’s precision. The accuracy of the method was formulated in terms of the percentage of model mixture recovery. The repeatability (intra-day) was evaluated by replication of eight measurements for three different CIL@LC-2a (1:1 wt./wt.) concentrations (low, 0.1 mg/mL; medium, 0.3 mg/mL; high, 0.4 mg/mL) and was performed on the same day using the proposed RP-HPLC method. Moreover, the intermediate precision (inter-day) was evaluated by comparing the results obtained from two different days for all concentrations of CIL@LC-2a (1:1 wt./wt.). 

Additionally, the limits of detection and quantification were evaluated. The limit of quantification (LOQ) and limit of detection (LOD) were calculated with the use of the following formulas (Equations (3) and (4)):

(3) $$\mathrm{LOD}=3{.3S}_{y}/a$$

(4) $$\mathrm{LOQ}={10S}_{y}/a$$

where Sy is the standard deviation of the blank signal and a is the slope of the calibration curve.

### 3.6. Stability Tests of Cilazapril and CIL@LC Particles

Kinetic studies were performed for CIL@LC-2a (1:1 wt./wt.). First, a forced ageing test was carried out. Samples of 20 mg of CIL@LC-2a (1:1 wt./wt.) were weighed, placed in open amber glass vials, and stored under the conditions described below. 

The effect of temperature was determined by preparing a series of samples which were then incubated at temperatures from 65 °C to 90 °C at RH ~76.4% (obtained using a bath of NaCl-saturated aqueous solution), in heat chambers in which the temperature was controlled with an accuracy of ±1.0 °C. 

The relative humidity (RH) effect was estimated under isothermal conditions within the RH range 25.0–76.4%. To obtain the desired level of RH, saturated salt baths were used: sodium iodide (RH ~ 25.0%), sodium bromide (RH ~50.9%), potassium iodide (RH ~60.5%), sodium nitrate (RH ~66.5%), and sodium chloride (RH ~76.4 %). For the stability tests, samples of CIL@LC-2a (1:1 wt./wt.) were introduced into an appropriate salt bath and placed in an automatically controlled heat chamber, which was set at 90 °C. Prior to the experiment, the salt baths had been incubated at the experiment temperature for 24 h to equilibrate the conditions of the kinetic test.

The concentration changes of CIL@LC-2a (1:1 wt./wt.) were determined based on the degradation rate of pure cilazapril. For this purpose, the vials were cooled to ambient temperature and dissolved in methanol (solution X). Then, 25 µL of the filtered solution was injected into the chromatographic column, and chromatograms were recorded. Kinetic curves were constructed using the method of least squares and were based on the remaining drug concentration *(c)* calculated from the measured relative peak areas *(Pi)*. Additionally, a methanolic solution of pure cilazapril at a concentration of 0.4 mg/mL was prepared for comparison (solution Y). Afterwards, 25 µm of the cilazapril solution was injected into the chromatographic column. 

Interpretation of the acquired data was performed using Equation (5):

(5) $$c_{\mathrm{CIL}\;}\left(\%\right)=f(t)$$

The initial concentration of cilazapril in pure in the samples (t = 0) was defined as 100%, and all measured concentrations were presented as percentages of the initial concentration. The content percentage loss was calculated as follows (Equation (6)):

(6) $$c\left(\%\right)=\left(P_{X}{\;\times \;c}_{B}\;\times \;V\right){\times (P}_{Y}\;\times \;m)$$

where $P_{X}$ is the magnitude of the cilazapril peak in solution X, $\;P_{Y}$ is the magnitude of the pure cilazapril peak in solution Y, $c_{B}$ is the percentage concentration of the comparative cilazapril in pure (c = 0.4 mg/mL), V is the volume coefficient (V = 25 mL), and *m* is the quantity of synthetic mixture (m = 20 mg).

The results were compared with those previously published for cilazapril in pure [38].

The Arrhenius relationship was used for calculation of activation energies (E_a_) (Equation (7)):

(7) $${\mathrm{lnk}}_{i}=\mathrm{lnA}-\frac{E_{a}}{\mathrm{RT}}$$

where $k_{i}$ is the reaction rate constant (1/s), $E_{a}\;\mathrm{is}\;\mathrm{the}\;\mathrm{activation}\;\mathrm{energy}\;(J/\mathrm{mol})\;$, *A* is the frequency coefficient, R is the universal gas constant (8.3144 J/(K·mol) and T is temperature.

Equations (8)–(10) were used to calculate the enthalpy of activation (${\Delta H\;}^{-}\;$) and entropy of activation (${\Delta S}^{-}\;$). The calculations were based on the transition state theory, under a temperature of 20 °C and relative humidity ~76.4%.

(8) $$E_{a}=-a\cdot R$$

(9) $$E_{a}={\Delta H}^{-}+\mathrm{RT}$$

where a is the slope of the straight line ${\mathrm{lnk}}_{i}=f(1/T)$, *A* is the frequency coefficient, R is the universal gas constant (8.3144 J/(K·mol), $E_{a}$ is the activation energy (J/mol), T is temperature (K), ${\Delta S}^{-}$ is the entropy of activation (J/(K· mol), ${\Delta H}^{-}$ is the enthalpy of activation (J/mol), K is Boltzmann’s constant, 1.3806488(13)·10^−23^ (J/K), and h is Planck’s constant, 6.62606957(29)·10^−34^ (J·s).

## 4. Conclusions

To summarize the present research, we obtained lignin-based spherical particles with the use of lignin and the cationic surfactant CTAB. Different ratios of precursors (1:1 wt./wt., 2:1 wt./wt. and 4:1 wt./wt.) were tested. Lignin-based particles were obtained at micro- and nanosizes, which was confirmed with the use of SEM images and evaluation of particle size distribution. Structures with the best morphological and dispersion properties were selected, and their acute toxicity was evaluated. The sample LC-2a (1:1 wt./wt.) exhibited the best overall parameters, and was thus selected for combination with an active compound, cilazapril, in the ratio 1:1. In physicochemical and morphological analysis, it was confirmed that lignin-based spherical particles were successfully combined with cilazapril. Electrokinetic and thermogravimetric analyses showed that the biopolymer particles significantly improved the stability of cilazapril. A more detailed stability test was carried out with the use of a reversed phase high-performance liquid chromatography method. The proposed RP-HPLC method was found to provide high efficiency, selectivity, linearity, sensitivity, and precision. Cilazapril degradation was performed according to first-order kinetics under increased humidity values and at increased temperatures. It was found that the activation energy of cilazapril in pure was much lower than that of the CIL@LC-2a (1:1 wt./wt.) structure. Moreover, combining LC-2a (1:1 wt./wt.) with CIL reduced the susceptibility of the tested drug to moisture.

The obtained LC-2a (1:1 wt./wt.) particles were non-toxic, their synthesis is simple and efficient, and the prepared material is characterized by a spherical shape. More importantly, the lignin-based spheres can be used as a protector of unstable active substances. Therefore, the results obtained are very promising and may form a basis for the further development of medical applications for lignin-based spherical particles.

## Figures and Tables

**Table 1:** Particle size distribution and polydispersity index for all obtained products.

Sample Name	Particle Size Distributions by Volume (nm)	Maximum Volume Contribution (%)	Polydispersity Index (PdI)
LC-2a (1:1 wt./wt.)	295–825	531 nm–28.3	0.186
LC-2a (2:1 wt./wt.)	220–531	342 nm–27.1	0.219
LC-2a (4:1 wt./wt.)	255–615	396 nm–29.1	0.330
LC-2b (1:1 wt./wt.)	255–615	396 nm–27.3	0.315
LC-2b (2:1 wt./wt.)	255–615	459 nm–30.8	0.111
LC-2b (4:1 wt./wt.)	106–1106 4145–6439	295 nm–9.2; 5560 nm–12.4	0.284
LC-4 (1:1 wt./wt.)	220–712	396 nm–27.8	0.238
LC-4 (2:1 wt./wt.)	255–825	396 nm–24.5	0.352
LC-4 (4:1 wt./wt.)	220–955	396 nm–20.8	0.218

**Table 2:** *A. fischeri* metabolism inhibition by LC-2a (1:1 wt./wt.), LC-2b (2:1 wt./wt.), LC-4 (1:1 wt./wt.) and LC-4 (2:1 wt./wt.).

Sample Name	*A. fischeri* Metabolism Inhibition	
	Effect After 5 min (%)	Effect After 15 min (%)
LC-2a (1:1 wt./wt.)	14	18
LC-2b (2:1 wt./wt.)	33	37
LC-4 (1:1 wt./wt.)	18	22
LC-4 (2:1 wt./wt.)	22	24

**Table 3:** Particle size distribution and polydispersity index for cilazapril (CIL), lignin-based spheres and cilazapril-lignin-based blend (CIL@LC (1:1 wt./wt.).

Sample Name	Particle Size Distributions by Volume (nm)	Maximum Volume Contribution (%)	Polydispersity Index (PdI)
CIL	38–68; 106–615	51 nm–12.1 164 nm–20.4	0.821
LC-2a (1:1 wt./wt.)	295–825	531 nm–28.3	0.186
CIL@LC-2a (1:1 wt./wt.)	164–3091	1718 nm–12.2; 1990 nm–12.2	0.363

**Table 4:** The effect of humidity on the stability of cilazapril in pure and CIL@LC-2a (1:1 wt./wt.) at 90 °C.

	Cilazapril in Pure	CIL@LC-2a (1:1 wt./wt.)
RH (%)	k ± Δk (1/s)	
25.0	(3.270 ± 0.241) 10^−6^	(7.564 ± 0.574) 10^−7^
50.9	(7.922 ± 0.964) 10^−6^	(1.545 ± 0.705) 10^−6^
60.5	(1.189 ± 0.068) 10^−5^	(1.999 ± 0.421) 10^−6^
66.5	(1.583 ± 0.143) 10^−5^	(2.367 ± 0.248) 10^−6^
76.4	(1.941 ± 0.106) 10^−5^	(3.883 ± 0.634) 10^−6^
linear relationship lnk = f(RH)		
a	0.0361 ± 0.0062	0.0304 ± 0.0068
SD_a_	0.0020	0.0024
b	−13.526 ± 0.231	−14.904 ± 0.462
SD_b_	0.102	0.143
r	0.997	0.990

Calculation of the parameters: a—slope, SD_a_—standard deviation of the slope, b—intercept, SD_b_—standard deviation of intercept, and k—correlation coefficient.

## Supplementary Materials

The following are available online. Figure S1: Particle size distributions of LC-2a (1:1 wt./wt.), LC-2a (2:1 wt./wt.), LC-2a (4:1 wt./wt.), LC-2b (1:1 wt./wt.), LC-2b (2:1 wt./wt.), LC-2b (4:1 wt./wt.), LC-4 (1:1 wt./wt.), LC-4 (2:1 wt./wt.) and LC-4 (4:1 wt./wt.), the sample was dispersed in propan-2-ol (Cp = 0.05%), Table S1: Validation parameters of the proposed method, Table S2: The thermodynamic and kinetic data for stability of cilazapril in pure and CIL@Lignin-CTAB-2a (1:1 wt./wt.).
