# Supplementary material for: Lignin-Based Spherical Structures and Their Use for Improvement of Cilazapril Stability in Solid State
Source: Molecules. 2020 Jul 9;25(14):3150. doi: 10.3390/molecules25143150 (PMC7397289; doi:10.3390/molecules25143150)
Supplement: Supplementary file 1 [file molecules-25-03150-s001.pdf]

Supplementary Materials:

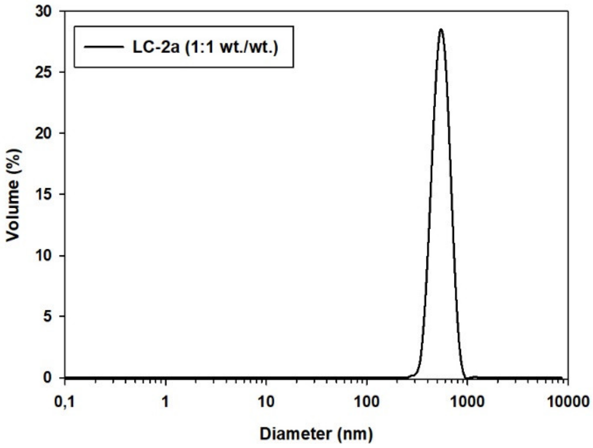

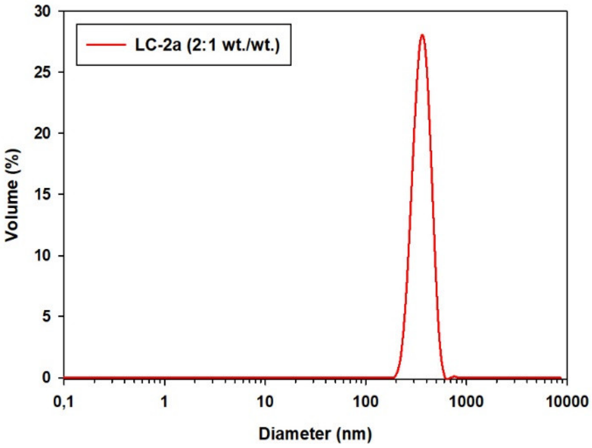

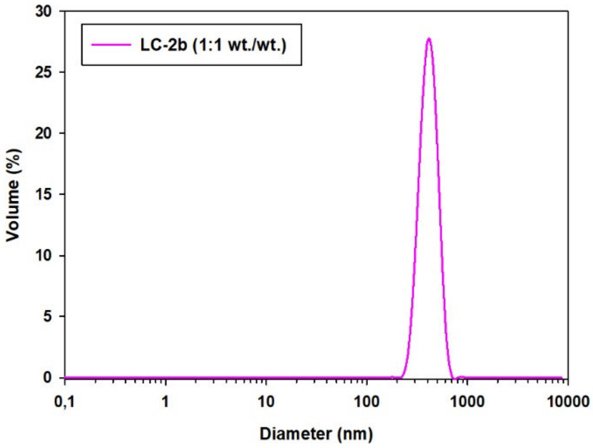

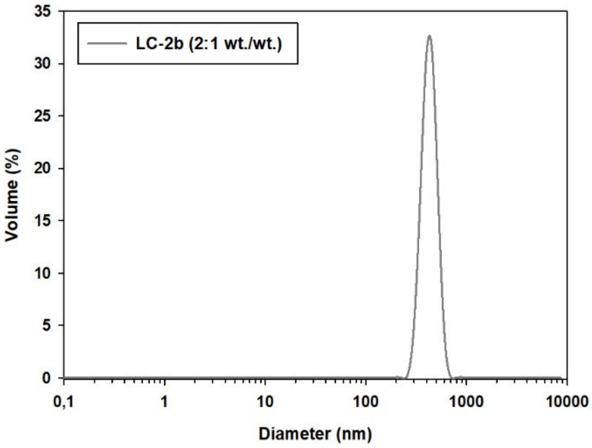

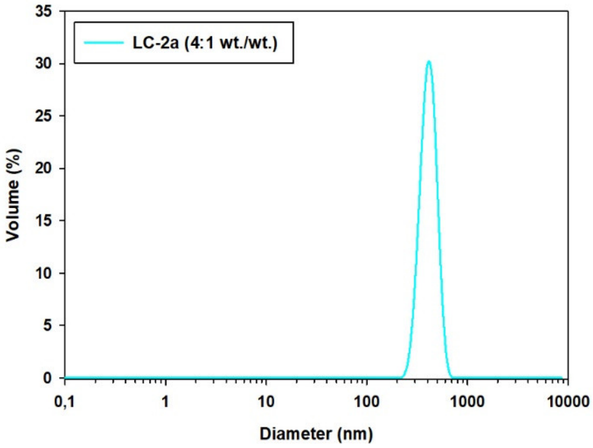

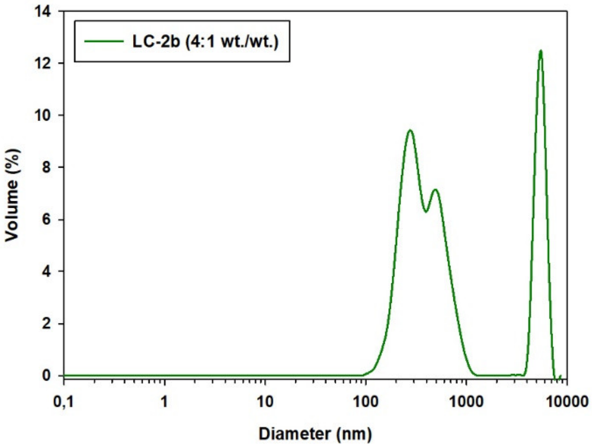

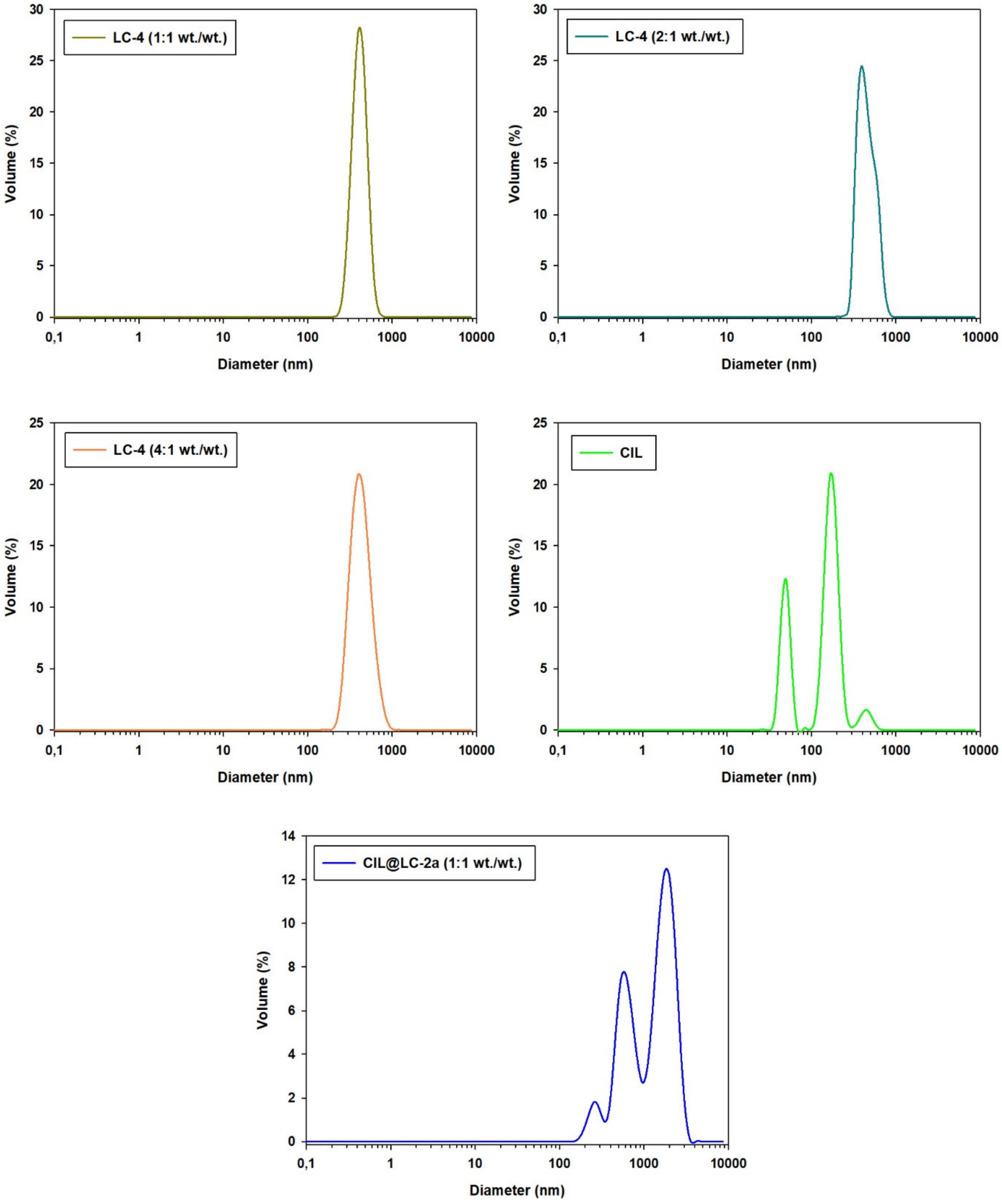

Figure S1. Particle size distributions of LC-2a (1:1 wt./wt.), LC-2a (2:1 wt./wt.), LC-2a (4:1 wt./wt.), LC-2b (1:1 wt./wt.), LC-2b (2:1 wt./wt.), LC-2b (4:1 wt./wt.), LC-4 (1:1 wt./wt.), LC-4 (2:1 wt./wt.) and LC-4 (4:1 wt./wt.). The sample was dispersed in propan-2-ol (Cp = 0.05%).

Table S1. Validation parameters of the proposed method.

Linearity parameters				
**Parameters**	**CIL@Lignin-CTAB-2a (1:1 wt./wt.)**		**CIL *in pure***	
Linearity range (mg/mL)	0.04 – 0.4		0.04 – 0.4	
Regression equations	y = ac + b			
Slope a ± Δa	4.842 ± 0.217		4.821 ± 0.247	
SD_a	1.017		1.019	
*Intercept b ± Δb	0.0209 ± 0.0067		0.0211 ± 0.0087	
SD_b	0.0222		0.0229	
Regression coefficient	0.999		0.999	
SD_y	0.0341		0.0361	
LOD (mg/mL)	0.023		0.025	
LOQ (mg/mL)	0.070		0.075	
**Precision and recovery data for the proposed method**				
	Concentration (mg/mL)	Amount found	% Recovery	Mean % Recovery ± SD
	Intra-day precision			
CIL@Lignin-CTAB-2a (1:1 wt./wt.)	0.1000	0.1001	100.10 ± 0.48	100.18 ± 0.49
	0.3000	0.3010	100.33 ± 0.99	
	0.4000	0.4004	100.10 ± 0.59	
	Inter-day precision			
	0.1000	0.1006	100.60 ± 0.36	100.20 ± 0.63
	0.3000	0.2999	99.96 ± 0.47	
	0.4000	0.4002	100.05 ± 0.45	

*intercept b from equation y = ac + b was statistically insignificant (t-Student test, α = 0.05); SD_a, SD_b, SD_y standard deviation of slope a, intercept b and y, respectively. The regression parameters: y = ac + b, a ± Δa, b ± Δb, the correlation coefficient r and standard errors SD_a and SD_b were calculated with a use of the least square's method.

Table S2. The thermodynamic and kinetic data for stability of cilazapril *in pure* and CIL@Lignin-CTAB-2a (1:1 wt./wt.).

**Temperature (°C/K)**	**k ± Δk (1/s)**	**r**	**Linear Arrhenius relationship f(1/T) = lnK**	**Thermodynamic parameters**
**Cilazapril *in pure***				
**65/338**	$(1.217 \pm 0.059)\ 10^{-7}$	-0.998	a = -20025.29 ± 2500.30	E_a = 166.49 ± 20.83 (kJ/mol)
**70/343**	$(7.607 \pm 0.418)\ 10^{-7}$	-0.998	s_a= 785.75	ΔH = 1664.02 ± 23.32 (kJ/mol)
**80/353**	$(1.662 \pm 0.129)\ 10^{-6}$	-0.994	b = 44.21 ± 7.22	ΔS = 122.68 ±185.11 (J/mol·K)
**85/358**	$(2.963 \pm 0.202)\ 10^{-6}$	-0.994	s_b = 2.26	
**90/363**	$(1.941 \pm 0.106)\ 10^{-5}$	-0.997	r = 0.998	
**CIL@Lignin-CTAB-2a (1:1 wt./wt.)**				
**65/338**	$(2.230 \pm 0.119)\ 10^{-9}$	-0.996	a = -36538.78 ± 7522.82	E_a = 303.79 ± 62.51 (kJ/mol)
**70/343**	$(2.259 \pm 0.406)\ 10^{-8}$	-0.996	s_a = 2709.96	ΔH = 306.26 ± 65.04 (kJ/mol)
**80/353**	$(6.199 \pm 0.701)\ 10^{-8}$	-0.992	b = 87.70 ± 21.45	ΔS = 484.25 ±66.98 (J/mol·K)
**85/358**	$(4.169 \pm 0.601)\ 10^{-7}$	-0.993	s_b = 7.72	
**90/363**	$(3.880 \pm 0.117)\ 10^{-6}$	-0.999	r = 0.991	

Parameters: k – degradation rate constants, $\Delta H^{\neq}$ – enthalpy of activation, $\Delta S^{\neq}$ – entropy of activation and E_a – energy of activation.
